# African Medicines Agency: How it will change the landscape of medicines in Africa

**DOI:** 10.1002/puh2.96

**Published:** 2023-06-06

**Authors:** Molly Unoh Ogbodum, Deborah Oluwaseun Shomuyiwa, Don Eliseo Lucero‐Prisno, Comfort Tanaka Gutu, Hajar Bouali, Buya Nabie Bangura, Moussa Fofana, Mohamed Babiker Musa, Hassan Ali Daoud, Priscilla Owusu‐Mensah, David Do Céu Fiagan, Cynthia Amaning Danquah, Mohamed Samai

**Affiliations:** ^1^ Department of Public Health University of Calabar Calabar Nigeria; ^2^ Global Health Focus Lagos Nigeria; ^3^ Faculty of Pharmacy University of Lagos Lagos Nigeria; ^4^ Department of Global Health and Development London School of Hygiene and Tropical Medicine London UK; ^5^ Faculty of Management and Development Studies University of the Philippines Open University Los Baños Laguna Philippines; ^6^ Faculty of Public Health Mahidol University Bangkok Thailand; ^7^ Ministry of Health and Child Care Harare Zimbabwe; ^8^ Faculty of Medicine and Pharmacy of Casablanca University Hassan II Casablanca Casablanca Morocco; ^9^ College of Medicine and Allied Health Sciences University of Sierra Leone Freetown Sierra Leone; ^10^ Université des Sciences des Techniques et des Technologies de Bamako Bamako Mali; ^11^ International Pharmaceutical Students Federation Bamako Mali; ^12^ Faculty of Pharmacy Omdurman Islamic University Khartoum Sudan; ^13^ Amoud University Borama Somalia; ^14^ Faculty of Pharmacy and Pharmaceutical Sciences Kwame Nkrumah University of Science and Technology Kumasi Ghana; ^15^ International Pharmaceutical Students’ Federation Ouagadougou Burkina Faso; ^16^ Université Joseph KI‐ZERBO Ouagadougou Burkina Faso; ^17^ Kwame Nkrumah University of Science and Technology Kumasi Ghana; ^18^ Department of Pharmacology and Therapeutics College of Medicine and Allied Health Sciences University of Sierra Leone Freetown Sierra Leone

**Keywords:** Africa, African Medicines Agency, health systems, medicines

## Abstract

The African continent, with a population of about 1.2 billion, faces limited access to safe, high‐quality and effective medicine, resulting in a disproportionate disease burden. However, the scarcity of pharmaceuticals has been a significant problem for decades. The need to scale up local production of medicines in Africa is apparent, as over 70% of drugs available in the continent are imported. Africa's pharmaceutical manufacturing industry capacity is subpar due to inadequate production equipment and substandard operations. Inadequate pharmaceutical supplies encouraged the circulation of fake drugs, and the COVID‐19 pandemic highlighted the dangers of the continent's reliance on foreign supplies. Africa's porous borders create ease for drug counterfeiting, with little likelihood of detection once in the supply chain. The African Medicines Agency (AMA) was founded to model the European Medicines Agency to enhance regional drug production, regulation and patient access. The African Union's AMA is a specialized health organization tasked with enhancing regulatory harmonization of medicines, particularly in pharmaceutical production, to increase access to high‐quality medications across the continent. Africa's healthcare industry, particularly domestic pharmaceutical manufacturing, will be a significant economic engine for the continent over the next 5 years. The establishment of AMA is a call to action for governments and regulators to enable Africa to manufacture 60% of the vaccines needed on the continent by 2040.

## INTRODUCTION

With 1.2 billion people, Africa is the second most populous continent after Asia [1]. Alongside limited access to high‐quality, cost‐effective, safe and effective medical products, the continent accounts for 60% of people living with HIV/AIDS and more than 90% of the annual global malaria cases [1]. The African continent has a significant 18.7% prevalence of substandard and counterfeit medications [1]. This is attributable to weakened or nonexistent medication regulatory systems, emphasizing the need for stringent and effective pharmaceutical product regulations [1, 2]. The scarcity of pharmaceuticals in Africa has been a problem for decades. Africa has few local and regional pharmaceutical manufacturing companies, with most production relegated to final fill‐and‐finish steps, packing and labelling within the region [2, 3]. In 2019, 70%–90% of drugs available in African pharmaceutical markets were imported, highlighting the continent's dependence on imported pharmaceutical goods to meet healthcare demands and needs [3].

**FIGURE 1:**
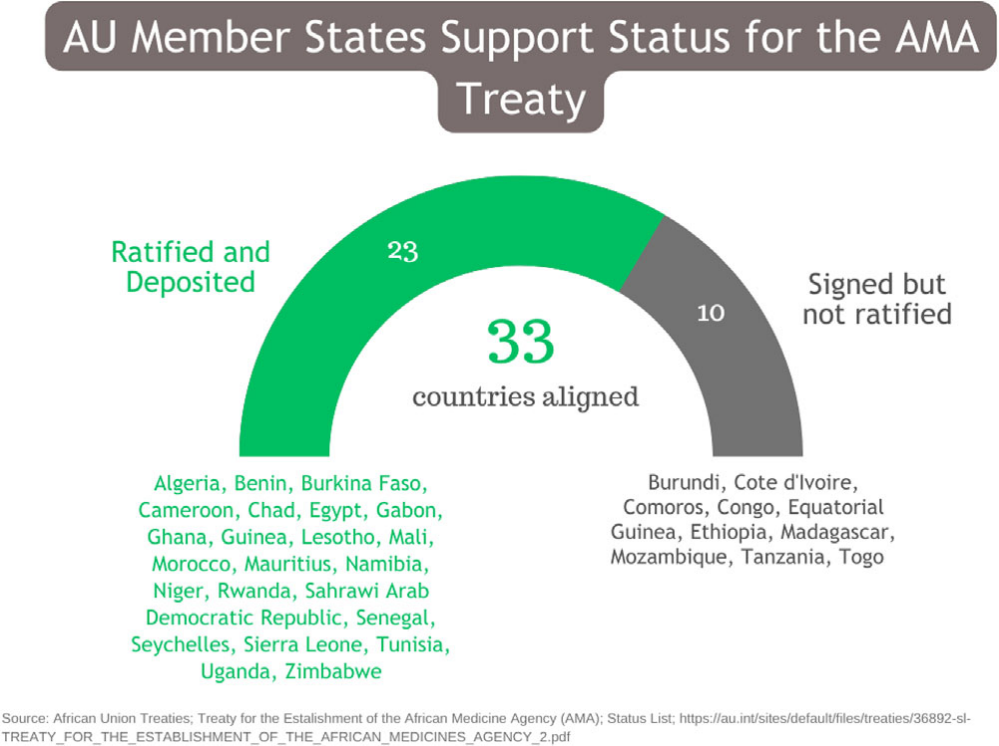
The status of African countries support to the African Medicines Agency treaty as of February 14, 2023.

Across Africa, local manufacturing and pharmaceutical companies are responding to the urgent need for locally produced medical products and technologies despite the existing regulatory challenges [4]. These challenges include weak legislative frameworks, duplicative and slow medicine registration processes, prolonged approval decisions, limited technical capacity and weak supply chain control [2, 4]. The African Medicines Agency (AMA), established on February 11, 2019, as a specialized agency under the African Union (AU), aims to improve the safety and efficacy of medical products by fostering regulatory alliances and harmonizing legislation across the continent [5]. Derived from the African Medicines Regulatory Harmonization (AMRH) initiative, which is led by the African Union Development Agency (AUDA‐NEPAD), the AMA has the potential to establish a unified regulatory system in Africa by addressing gaps in national frameworks and mobilizing technical assistance and resources at a continental level [1, 4, 5]. As of February 14, 2023, 33 member states of the AU have signed or ratified the AMA treaty, which came into effect on November 5, 2021 (Figure 1) [6]. This article evaluates the challenges of accessing medicine in Africa and assesses the potential impact of the AMA on the future of medicine and health systems across the continent.

## ISSUES AFFECTING MEDICINES MANUFACTURING AND ACCESS IN AFRICA

As of 2020, there were approximately 600 pharmaceutical manufacturers in Africa, compared to 5000 and 10,500 in China and India, respectively [3]. This highlights Africa's reliance on imports as several interrelated barriers prevent domestic production [3]. These imports are mainly from Europe, India, Switzerland, China, the United States and the United Kingdom [3, 7]. However, the COVID‐19 pandemic exposed the risk of relying on foreign suppliers of pharmaceutical products, as the shortage of medicine led to a surge in counterfeit production [6]. Counterfeiting is a relatively easy act in Africa due to porous borders with little chance of being detected or caught once introduced to the supply chain [8]. Consequently, the impact of the COVID‐19 pandemic underscores the need for a robust regulatory framework that strengthens domestic manufacturing capacity for pharmaceutical products and medical supplies [8].

Following small manufacturing capacities, limited production equipment and operations that do not meet international standards, the state of the art of pharmaceutical manufacturing industries is below par in Africa. This is detrimental to population health as the focus on imports accrues high medical costs and expenses, affecting affordability among users [7]. In addition, the quality of manufactured pharmaceuticals is significantly influenced by the availability of raw materials, particularly active pharmaceutical ingredients. However, most raw materials and equipment required for drug production are imported, leading to high production costs [7]. The shortage of well‐trained and skilled researchers is common in African medical schools and health institutions. The region needs more research to support policies’ design, development and implementation to effectively address its diverse health challenges [8]. Without adequate research, it is impartial to make significant strides in developing and innovating health policies that can improve health outcomes on the continent.

Access to medicines and healthcare also remains challenging in Africa due to dependency on Western donor funding grounded on weak sustainable health financing, thus limiting the optimization of quality health among the vast majority [7]. This dependency was exposed during the advent of COVID‐19 and the Ebola outbreak, revealing the vulnerability and risk of the continent's reliance [8]. Local drug production is further plagued by restrictive policies, weak economies and high poverty rates [7]. In most African countries, the absence of critical infrastructure, such as transportation and communication networks and reliable power supplies, impeded the development of regional pharmaceutical enterprises [8]. To improve domestic pharmaceutical systems, all building components of health systems, including policy, regulation, innovation, research and development, manufacturing, financing and human resources, must be strengthened.

African healthcare systems continue to be characterized by disparities in the three‐tiered parallel systems of public, private and traditional healthcare, presenting one of the most significant challenges for governments to ensure equal and equitable access to healthcare [8]. Despite the continent accounting for 24% of the global disease burden, African nations produce only 3% of global drug production, underscoring the struggle to provide equitable access to medicines and healthcare to all its population [7]. The constant focus on achieving economic objectives and budget cuts to social services continue to undermine healthcare delivery and exacerbate access inequalities [7, 8]. By supporting the supply of affordable and efficient medications, particularly for neglected diseases that disproportionately impact the poor, the AMA will ensure equitable access to medicines across African countries. Additionally, the agency will assist in enhancing the capability of national regulatory agencies and the regulatory climate for Africa's pharmaceutical sector [9].

## UNLOCKING ECONOMIC GROWTH AND ACHIEVING HEALTH SECURITY IN AFRICA WITH AMA

Encouraging local production is believed to be an effective way to increase access to medicines in Africa as it would shorten the supply chain, reduce import costs and aid in developing the industrial complex on the continent [9]. The AMA was established to model the European Medicines Agency (EMA) and improve Africa's medicine production, regulation and patient access. Amongst numerous roles, coordination and cooperation with national medicines regulatory agencies are one of the EMA's recognized roles [10]. It also promotes communication and a more comprehensive exchange of information, improving pharmaceuticals’ quality, safety and effectiveness worldwide [10]. While emulating the EMA, experiences from other resource‐constraint countries and regional initiatives such as the South‐East Asia Regulatory Network (SEARN) can ease contextualization, especially for pharmacovigilance. AMA must also liaise, collaborate and learn from the experiences of other similar agencies within the African region.

The AMA is tasked with improving the regulatory harmonization of medicines, notably in pharmaceutical manufacturing, to improve access to quality‐assured drugs across the continent. Aligning technical requirements for developing and marketing medicines across the countries will build a unified regulatory framework and develop regulatory competence, strengthen governance and build collaboration, emphasizing continual improvement and transparency. Given the selection of Rwanda as a host country, it must now set up AMA's board and mobilize resources [11]. The agency will coordinate and strengthen medicine regulatory harmonization initiatives across the continent, examine regional policies, find new financing sources and make an effort to simplify the complex requirements from regional and international standards and recommendations [1, 4, 11].

Some academics have drawn attention to the debate on how local drug production is polarized by the unsolved conflict between industrial policy goals and public health concerns [9]. However, by mobilizing technical assistance and resource sharing at a level neither national nor regional initiatives can match, the AMA can defeat these conflicts and promote harmonization [1, 11]. Harmonizing health product regulations will align Africa's fragmented markets and make Africa become a more desirable market for the pharmaceutical industry, both for R&D and the introduction of innovations [8]. These harmonization initiatives will further enhance trade support of the African Continental Free Trade Area (AfCFTA). Most importantly, the agency will organize capacity‐building, collaborative assessments and inspections for certain items [4, 11].

The goal is to gradually switch from a country‐focused strategy to a cooperative regional strategy in which five Regional Economic Communities support one agency to improve the continent's disjointed regulatory system for the registration of medical products [4]. Over the next 5 years, Africa's healthcare sector, especially local pharmaceutical production, will be a critical economic driver for the region [11]. The founding of AMA is a rallying cry to nations and regulators, with the goal of preparing Africa to enable the manufacture of 60% of the vaccinations required on the continent by 2040. The instruments the agency needs to be fully operational by the end of 2022 must be immediately put in place [4, 11]. Governments, regulatory systems, manufacturers and relevant stakeholders must collaborate and productively play their roles in Africa's journey towards providing safe, quality and effective medicine for its people. Optimizing regulatory reliance by leveraging the outputs/assessments conducted by other regulatory authorities can help save resources, increase efficiency and avoid duplication in regulatory pathways. Encouraging increased government funding and private sector investment will work on the challenge of weak financing of medicines production [12]. Investment in technological advancement in health and medical science results in higher productivity and improves population health outcomes.

## CONCLUSION

The AMA has a rare opportunity to develop into one of the world's most influential and cutting‐edge regulatory systems. The challenges of weak financing, counterfeiting, excessive reliance, lack of efficient regulatory bodies and clinical research have plagued the region over time. Africa has started the long walk to provide safe, quality and effective medicines for its people. Hence, governments, regulatory systems, manufacturers and relevant stakeholders must work together and play their roles in making this a reality. Therefore, all interested parties must work cooperatively and assist the AMA establishment within their spheres of influence across the continent and beyond.

## AUTHOR CONTRIBUTIONS

Writing – original draft; writing – review and editing: Molly Unoh Ogbodum. Project administration; writing – original draft; writing – review and editing: Deborah Oluwaseun Shomuyiwa. Conceptualization; project administration; writing – original draft; writing – review and editing: Don Eliseo Lucero‐Prisno III. Writing – review and editing: Comfort Tanaka Gutu, Hajar Bouali, Buya Nabie Bangura, Moussa Fofana, Mohamed Babiker Musa, Hassan Ali Daoud, Priscilla Owusu‐Mensah, David Do Céu Fiagan, Cynthia Amaning Danquah and Mohamed Samai.

## CONFLICT OF INTEREST STATEMENT

Deborah Oluwaseun Shomuyiwa is a Youth Editorial Board member of Public Health Challenges and a co‐author of this article. Cynthia Amaning Danquah is an Editorial Board member of Public Health Challenges and a co‐author of this article. Don Eliseo Lucero‐Prisno III is the Editor‐in‐Chief of Public Health Challenges. To minimize bias, they have been excluded from all editorial decision‐making related to the acceptance of this article for publication.

## FUNDING INFORMATION

There is no funding for the development of this paper.
